# *Plasmodium falciparum* coronin organizes arrays of parallel actin filaments potentially guiding directional motility in invasive malaria parasites

**DOI:** 10.1186/s12936-015-0801-5

**Published:** 2015-07-18

**Authors:** Maya A Olshina, Fiona Angrisano, Danushka S Marapana, David T Riglar, Kartik Bane, Wilson Wong, Bruno Catimel, Meng-Xin Yin, Andrew B Holmes, Friedrich Frischknecht, David R Kovar, Jake Baum

**Affiliations:** Infection and Immunity Division, Walter and Eliza Hall Institute of Medical Research, Parkville, VIC 3052 Australia; Department of Medical Biology, University of Melbourne, Parkville, VIC 3052 Australia; Department of Infectious Diseases, University of Heidelberg Medical School, Heidelberg, Germany; Ludwig Institute for Cancer Research, Melbourne Tumour Biology Branch, Royal Melbourne Hospital, Parkville, VIC 3052 Australia; School of Chemistry, Bio21 Institute, University of Melbourne, Parkville, VIC 3010 Australia; Department of Molecular Genetics and Cell Biology, The University of Chicago, Chicago, USA; Department of Biochemistry and Molecular Biology, The University of Chicago, Chicago, USA; Department of Life Sciences, Imperial College London, Sir Alexander Fleming Building, Level 6, South Kensington, London, SW72AZ UK; Department of Systems Biology, Harvard Medical School, 200 Longwood Ave WAB 536, Boston, MA 02115 USA; Systems Biology and Personalised Medicine Division, Walter and Eliza Hall Institute of Medical Research, Parkville, VIC 3052 Australia

**Keywords:** Gliding motility, Coronin, Actin, *Plasmodium*, Tight junction, Merozoite

## Abstract

**Background:**

Gliding motility in *Plasmodium* parasites, the aetiological agents of malaria disease, is mediated by an actomyosin motor anchored in the outer pellicle of the motile cell. Effective motility is dependent on a parasite myosin motor and turnover of dynamic parasite actin filaments. To date, however, the basis for directional motility is not known. Whilst myosin is very likely orientated as a result of its anchorage within the parasite, how actin filaments are orientated to facilitate directional force generation remains unexplained. In addition, recent evidence has questioned the linkage between actin filaments and secreted surface antigens leaving the way by which motor force is transmitted to the extracellular milieu unknown. Malaria parasites possess a markedly reduced repertoire of actin regulators, among which few are predicted to interact with filamentous (F)-actin directly. One of these, PF3D7_1251200, shows strong homology to the coronin family of actin-filament binding proteins, herein referred to as PfCoronin.

**Methods:**

Here the N terminal beta propeller domain of PfCoronin (PfCor-N) was expressed to assess its ability to bind and bundle pre-formed actin filaments by sedimentation assay, total internal reflection fluorescence (TIRF) microscopy and confocal imaging as well as to explore its ability to bind phospholipids. In parallel a tagged PfCoronin line in *Plasmodium falciparum* was generated to determine the cellular localization of the protein during asexual parasite development and blood-stage merozoite invasion.

**Results:**

A combination of biochemical approaches demonstrated that the N-terminal beta-propeller domain of PfCoronin is capable of binding F-actin and facilitating formation of parallel filament bundles. In parasites, PfCoronin is expressed late in the asexual lifecycle and localizes to the pellicle region of invasive merozoites before and during erythrocyte entry. PfCoronin also associates strongly with membranes within the cell, likely mediated by interactions with phosphatidylinositol-4,5-bisphosphate (PI(4,5)P_2_) at the plasma membrane.

**Conclusions:**

These data suggest PfCoronin may fulfil a key role as the critical determinant of actin filament organization in the *Plasmodium* cell. This raises the possibility that macro-molecular organization of actin mediates directional motility in gliding parasites.

**Electronic supplementary material:**

The online version of this article (doi:10.1186/s12936-015-0801-5) contains supplementary material, which is available to authorized users.

## Background

The infectious stages of *Plasmodium* parasites, the etiological agents of malaria, are exquisitely designed for cell motility and targeted host cell invasion. Despite traversing several very different tissue environments and invading divergent host cells in the mosquito and vertebrate hosts [1, 2], each life cycle stage retains a conserved cellular architecture [3, 4] and mode of actomyosin-based cell movement, called gliding motility. The mechanics of gliding is intimately linked to the cellular architecture of the apicomplexan cell, the phylum to which malaria parasites belong [4]. The pellicular region underlying the plasma membrane houses much of the machinery associated with motility. Comprised of a series of membrane bound structures, termed the inner membrane complex or IMC, this compartment lies some 20–50 nm under the plasma membrane [4], the space between housing the gliding motor complex. Current models predict that this structure is composed of a single headed myosin motor, anchored between the IMC and plasma membrane via a series of binding partners [5]. Motor action then provides the requisite force necessary for motility [6]. Myosin engages directly with transient, short actin filaments that are themselves linked to the extracellular milieu via the cytoplasmic tails of surface bound adhesins [7]. Until recently, the linkage between actin filaments and the terminal residues of surface-bound adhesins was thought to involve the glycolytic enzyme fructose 1,6 bisphosphate aldolase [7], which is known to bind actin [8]. According to this model, directional passage of the actin-aldolase-adhesin complex rearwards by the myosin motor would then generate rearward force driving the parasite forwards.

Whilst the current model for gliding is appealing it does not explain how directional motility is generated. To date, efforts to visualize motor organization or actin in the pellicle of the parasite have not been successful [9, 10]. Recent evidence from the apicomplexan parasite *Toxoplasma gondii,* a distant relative of *Plasmodium* parasites, has also brought into question not only the role of aldolase, beyond its metabolic contribution [11], but also the essential contribution of each motor complex component to motility in general [12]. Thus, the question of how directional force generation and subsequent directional gliding is achieved remains entirely unresolved.

Drugs that perturb microfilament turnover demonstrate the importance of dynamic actin to gliding motility [13, 14]. Since actin forms a polar filament, proteins that interact in an orientated fashion constitute attractive candidates that might impact significantly on directional motility. Computational analysis of available apicomplexan genomes has revealed a remarkable reduction in the repertoire of identifiable actin regulators and actin-binding proteins in this phylum [15–17]. The minimal set in *Plasmodium* parasites includes two formins [18], a single profilin [19], two actin depolymerizing factors (ADF) [20, 21], a homologue of the yeast-actin regulator srv2 or CAP protein [22, 23], capping subunits alpha and beta [24] and coronin [25]. Among this minimal repertoire of core actin regulators only coronin appears to fulfil the role of a specific filament binding protein. Coronins are a family of proteins implicated in several roles involving actin dynamics across eukaryotic systems including filament binding and bundling [26–28]. Based on sequence comparison coronins have been divided into three groups, Types I-III [26]. Type I coronins are defined by a three-part structure consisting of an N terminal seven bladed beta-propeller motif composed of WD40 repeats [29], a ‘‘unique’’ middle region that varies in sequence and length between variants and species, and a C-terminal coiled-coil (CC) domain that mediates homo-oligomerization [30] and interactions with the Arp2/3 complex [31], a key nucleation complex entirely absent in malaria parasites. Apicomplexan coronins are predicted to resemble Type I coronins, with recent structural analysis of *T. gondii* coronin (TgCoronin) revealing a conserved N-terminal beta propeller motif and mostly conserved actin-binding residues, as well as a potential dimerization motif in the C-terminal CC domain [32].

Here the biochemical interactions of the *Plasmodium falciparum* coronin-like protein (PfCoronin) and actin were addressed towards dissecting its role in organizing directional actin-based motility in *Plasmodium* parasites. Using genetic, cellular, biochemical and single molecule approaches, we show that PfCoronin is a true actin-filament binding protein able, in vitro, to direct filamentous (F)-actin into parallel bundles. PfCoronin peak expression is centred in late schizogony, where the protein localizes to the merozoite pellicle throughout invasion consistent with a role in motility. PfCoronin interacts with membrane fractions of the parasite cell, likely binding via phosphatidylinositol 4,5-bisphosphate (PI(4,5)P_2_). Given its potential for organizing actin filaments in situ at the parasite periphery into parallel bundles, a model in which coronin is a key determinant of directional gliding motility in apicomplexan parasites can be proposed.

## Methods

### Cloning, protein expression and purification

*PfCoronin:* The full-length gene encoding 6×His-PfCoroninFL was codon optimized for expression in Sf21 cells (GeneArt). The synthetic gene was cloned into the pFastBacHTB vector using BamHI/XhoI restriction sites. Bacmid DNA was produced according to the Bac-to-Bac manual (Invitrogen) using MultiBac cells. Bacmid DNA was transfected into Sf21 cells using Cellfectin II (Invitrogen) according to the manufacturers instructions. Viral stocks were amplified and used at a 1:1,000 dilution for protein expression. Following addition of virus, Sf21 cells were incubated in suspension at 27**°**C and harvested by centrifugation after 72 h. Cells were re-suspended in lysis buffer (50 mM Tris pH 8.0, 300 mM NaCl, 10 mM MgCl_2_, 5 mM 2-mercaptoethanol, 0.5% Triton X-100) supplemented with cOmplete EDTA-free protease inhibitors (Roche) and subjected to two rounds of freeze–thaw in liquid N_2_. The lysate was incubated with 1 mg/mL DNAseI for 30 min rocking at 4**°**C, followed by centrifugation at 30,000*g* for 30 min. The soluble fraction was recovered, adjusted to 10 mM imidazole pH 8.0 and incubated with Profinity™ IMAC resin for 2 h at 4**°**C. The resin was washed sequentially with buffer 1 (50 mM Tris pH 8.0, 300 mM NaCl, 5 mM 2-mercaptoethanol, 20 mM Imidazole), buffer 2 (50 mM Tris pH 8.0, 1 M NaCl, 5 mM 2-mercaptoethanol) and buffer 1. Protein was eluted in elution buffer (50 mM Tris pH 8.0, 300 mM NaCl, 5 mM 2-mercaptoethanol, 250 mM Imidazole) and analysed by SDS PAGE. Fractions containing His-PfCoronin were pooled and cleaved overnight with TEV protease during dialysis against buffer 3 (50 mM Tris pH 8.0, 300 mM NaCl, 5 mM 2-mercaptoethanol) to remove the 6×His tag. The dialyzed protein was incubated with Profinity™ IMAC resin for 2 h to bind un-cleaved protein. The cleaved protein was collected and the resin washed with buffer 3 until no more protein came off as monitored by Bradford reagent (BioRad). The cleaved sample and washes were pooled and concentrated to 0.5 mL. The protein was subjected to size exclusion chromatography on a Superdex 200 10/300 GL column (GE Healthcare) pre equilibrated in 30 mM Tris pH 8.0, 300 mM NaCl, 5 mM 2-mercaptoethanol. Full length PfCoronin eluted at ~12 mL. The N-terminal breakdown product PfCoronin 1-388 (called herein PfCor-N), eluted at ~16 mL. Peak fractions were analysed by Coomassie-stained SDS PAGE to assess protein purity. Fractions containing PfCor-N were pooled, concentrated and stored at 4**°**C.

*PfAldolase* The gene for PfAldolase was amplified from *P. falciparum* genomic DNA using the primers PfAldoF 5′GATCGGATCCATGGCTCATTGCACTGAATATATG and PfAldoR 5′GATCCTCGAGTTAATAGACATATTTCTTTTC, and ligated into the pProEX-HTb vector (Invitrogen) via BamHI/XhoI restriction sites, introducing an N-terminal 6×His tag. The plasmid was transformed into BL21 (DE3) *Escherichia coli* cells and the protein expressed for 4 h at 37**°**C after addition of 1 mM IPTG. The cells were harvested, re-suspended in lysis buffer (20 mM Tris pH 8.0, 300 mM NaCl, 0.3% Triton X-100, 5 mM 2-mercaptoethanol) supplemented with cOmplete EDTA-free protease inhibitors. The suspension was sonicated and clarified by centrifugation at 30,000*g* for 30 min at 4**°**C. The supernatant was collected, adjusted to 10 mM imidazole pH 8.0 and incubated for 2 h at 4**°**C with Profinity ™ IMAC resin. The resin was washed sequentially with Wash Buffer 1 (50 mM Tris pH 8.0, 300 mM NaCl, 20 mM imidazole pH 8.0, 5 mM 2-mercaptoethanol), Wash buffer 2 (50 mM Tris pH 8.0, 1 M NaCl, 5 mM 2-mercaptoethanol) and Wash Buffer 3 (50 mM Tris pH 8.0, 300 mM NaCl, 5 mM 2-mercaptoethanol). His-PfAldolase was eluted with elution buffer (Wash Buffer 3 + 250 mM imidazole pH 8.0) and assessed for purity and quantity by SDS PAGE. Elution fractions containing His-PfAldolase were pooled and dialysed against Buffer A (50 mM MES pH 7.0, 100 mM NaCl, 2 mM DTT, 1 mM EDTA) for 2 h, then subjected to cation exchange chromatography using HiTrap SPFF (GE Healthcare). A gradient from buffer A to buffer B (50 mM MES pH 7.0, 1 M NaCl, 2 mM DTT, 1 mM EDTA) was used to elute the protein. Peak fractions containing His-PfAldolase, as determined by Coomassie-stained SDS PAGE, were pooled, concentrated and subjected to size exclusion chromatography using a Superdex 200 10/300 gel filtration column (GE Healthcare) pre-equilibrated in Buffer A. His-PfAldolase eluted off the column as a single peak at ~13 mL, corresponding to a molecular weight of ~160 kDa which approximates the size of a tetramer. Peak fractions were pooled, concentrated to 100 uM, aliquoted, flash frozen in liquid N_2_ and stored at −80**°**C. Actin was purified from rabbit skeletal muscle acetone powder (Sigma-Aldrich) using established protocols [33].

### Sedimentation assays

*High speed* 2 μM RSMA in CaBG was adjusted by the addition of 10× Mg-EGTA exchange buffer (ME) (10 mM MgCl_2_, 2 mM EGTA) to make Mg bound RSMA (Mg-ATP-Actin). Mg-ATP-Actin was polymerized by the addition of 10× KMEI (0.5 M KCl, 0.1 M imidazole pH 7.0, 0.01 EGTA pH 8.0, 0.01 M MgCl_2_) and incubation for 2 h at room temperature. Proteins of interest [PfCor-N, PfAldolase and alpha-Actinin (Cytoskeleton Inc.)] were added to the appropriate concentration and the mixture incubated for a further 30 min at room temperature. The samples were centrifuged at 60,000 rpm in a Beckman preparative ultracentrifuge for 1 h at room temperature. The supernatant was carefully removed and adjusted with 5× RSB. The pellet was rinsed with MgBG (2 mM Tris pH 8.0, 0.2 mM ATP, 0.5 mM DTT, 0.1 mM MgCl_2_) and centrifuged at 60,000 rpm in a Beckman preparative ultracentrifuge for 1 h at room temperature. The supernatant was carefully removed and discarded, and the pellet re-suspended in 2× RSB to a volume equivalent to the first supernatant after addition of RSB. The supernatant and pellet samples were boiled for 5 min and equal volumes were separated by SDS PAGE, the gels stained with Coomassie brilliant blue (BioRad) and the bands analysed by densitometry.

*Low speed* Low-speed sedimentation assays were performed as per high-speed sedimentation assays with the following alterations. Mg-ATP-Actin was polymerized in the presence of the proteins of interest for 2 h at room temperature. Samples were centrifuged at 13,000 rpm in a standard benchtop centrifuge at 4**°**C. Supernatants were carefully collected and the pellet discarded. Supernatants were adjusted with 5× RSB, boiled for 5 min and separated by SDS PAGE. The gels were stained with Coomassie brilliant blue and the bands analysed by densitometry.

*Kd determination* Pre-polymerized Mg-ATP-Actin, prepared as per the high-speed sedimentation assay protocol, was incubated with the protein of interest for 30 min then centrifuged at 60,000 rpm for 1 h at 22°C. The supernatants were collected, and adjusted with 5× RSB. The pellets were rinsed with 1× KMEI and centrifuged as per the high-speed sedimentation assay protocol. The pellets were re-suspended in 2× RSB to the equivalent volume of the supernatant samples. Equal volumes were separated by SDS PAGE and the gels stained with Coomassie brilliant blue. For assays involving proteins too close in size to resolve by standard Coomassie staining, following SDS PAGE the proteins were subjected to Western blot analysis. Band densities were analysed by densitometry and *Kd*s determined according to the methods outlined in [34, 35].

### Electron microscopy

Appropriate amounts of purified PfCor-N or PfAldolase were added to 2 μM preformed Mg-ATP-Actin filaments for 30 min at room temperature. The samples were adsorbed onto Formvar-carbon films supported on 200-mesh copper grids. Grids were glow discharged before sample application, then negatively stained with aqueous uranyl acetate (1%). Samples were observed with an FEI Tecnai F30 microscope at 300 kV.

### Fluorescence microscopy

Mg-ATP-Actin was polymerized by the addition of 2× TIRF buffer alone or in the presence of proteins of interest and incubated in a covered tube at room temperature for 1 h. The samples were incubated with 1 μM Alexa Fluor ^®^ 488 Phalloidin (Life Technologies) for 5 min at room temperature. 3 μL of the samples were adsorbed onto coverslips coated with 0.05 μg/μL poly-l-Lysine (Sigma-Aldrich). Fluorescence images were acquired using a Zeiss inverted LSM-510 confocal microscope and processed using ICY image analysis software [36].

### TIRF microscopy

Oregon Green (OG) labelled RSMA was prepared as previously described [37]. 1.5 μM Mg-ATP-Actin (33% OG labeled) alone and in the presence of proteins of interest (Pf-Cor or Fimbrin, a kind gift from Colleen T. Skau) was prepared for TIRF microscopy by the addition of 2× TIRF buffer (10 mM imidazole pH 7.0, 50 mM KCl, 5 mM MgCl_2_, 1 mM EGTA, 0.5 mM DTT, 0.2 mM ATP, 50 μM CaCl2, 15 mM glucose, 20 μg/mL catalase, 100 μg/mL glucose oxidase, 0.5% methylcellulose 400 cP) to stimulate polymerization. The samples were immediately loaded into a pre-made flow chamber and excited by evanescent wave fluorescence on an IX-71 Olympus microscope fit with through the objective TIRF illumination. Images were acquired every 15 s for 10–20 min by an iXon EMCCD camera (Andor Technology) as previously described [38]. Movies were processed and analysed using ImageJ.

### *Plasmodium falciparum* culture and maintenance

The 3D7 *P. falciparum* isolate was cultured as previously described [39]. Parasites were maintained in O^+^ erythrocytes (Australian Red Cross Blood Bank, South Melbourne, Australia) at approximately 4% haematocrit, in a culture medium of RPMI-HEPES supplemented with 0.18% (w/v) NaHCO3 and 10% (v/v) pooled human serum from unexposed Melbourne blood donors or 0.5% (w/v) AlbumaxII (Gibco). Cultures were incubated at 37°C under a 94% N_2_, 1% O_2_, 5% CO_2_ gas environment. Transfected lines were maintained in the presence of appropriate drugs to select for the corresponding resistance marker included in the transfection vectors.

### Reverse transcriptase PCR (RT-PCR)

RT-PCR was performed as described [40]. Briefly, total RNA was extracted from synchronized 3D7 parasites at appropriate time points post-invasion using TRIzol ^®^ (Invitrogen), residual genomic DNA was removed using an RNAeasy ^®^ column (Qiagen), and 5 μg of total RNA was reverse transcribed with or without SuperScript ™ II reverse transcriptase using random hexamers (Invitrogen), all according to the manufacturers instructions. The following primers were used: Cor_RT_fwd (5′-CCTTTAATCAAGAATTTATA-TCC-3′) and Cor_RT_rev (5′-CCTCATTCACATTCTCATCCTC-3′); ACT1_RT_fwd (5′-CCAAAGAATCCAGGAATTATGG-3′) and ACT1_RT_rev (5′-GGAACAGTGTGTGATA-CACCATC-3′).

### Vector construction and tagging

Endogenous tagging of PfCoronin (PF3D7_1251200) at the C-terminus was performed as described using the pD3HA vector with parasite transfection following standard protocols [41].

### Antisera and immunoprecipitation

Antisera was raised in rabbits against PfCor-N, expressed and purified from BL21 (DE3) *E. coli* using standard methods. Immunoprecipitation was performed as previously described [40]. Briefly, 40–48 h 3D7 PfCoroninHA schizonts were subjected to protein extraction using 1% TNET (1% Triton X-100, 50 mM Tris pH 7.4, 150 mM NaCl, 5 mM EDTA) supplemented with cOmplete EDTA-free protease inhibitor cocktail (Roche). Pull downs were performed using anti-PfCoronin or anti-HA coupled to protein G-Sepharose (Amersham Biosciences) according to the manufacturers instructions. Proteins were separated by SDS PAGE and subjected to western blot analysis. The blots were probed with rat anti-HA [1:1,000] or rabbit anti-PfCoronin [1:1,000] and processed as previously described.

### Immunofluorescence assays

Parasites were synchronized according to established methods to obtain late schizonts or merozoites for re-invasion using either sorbitol or heparin treatment [42, 43]. Schizonts or invading parasites were fixed in a fixing solution of 4% paraformaldehyde (ProSciTech)/0.0075% gluteraldehyde (ProSciTech) in phosphate buffered saline (PBS) while rocking at room temperature for 30 min. Cells were permeabilized using 0.1% Triton X-100 (BioRad) for 10 min at room temperature and blocked overnight using Blocking Solution [3% (w/v) Bovine Serum Albumin (BSA) (Sigma-Aldrich) in PBS], while rocking at 4°C. Cells were incubated with appropriate primary antibodies diluted in Blocking Solution for 1 h at 4°C. Primary antibodies used were rat anti-HA [1:1,000] (Roche), rabbit anti-PfGAP45 [1:500] [40], rabbit anti-Act 239-253 [1:300] [10], mouse anti-PfRON4 [1:500] [44]. Samples were washed twice in PBS and incubated for 1 h at 4°C with appropriate secondary antibodies: Alexa Fluor ^®^ 488 or 594 goat anti-mouse, Alexa Fluor ^®^ 488 or 594 goat anti-rabbit and Alexa Fluor ^®^ 594 goat anti-rat (Invitrogen) [1:500] in Blocking Solution. Samples were washed three times in PBS and cells were settled onto coverslips (type 1.5, Zeiss) coated with 1% polyethyleneimine (PEI) (Sigma-Aldrich). Cells were mounted with VectaShield ^®^ (Vector Laboratories) with 0.1 ng/μL 4′,6-diamidino-2-phenylindole (DAPI) (Invitrogen). Fluorescence images were acquired using Plan-Apochromat 100×/1.40 oil immersion Phase contrast lens (Zeiss) on an AxioVert 200 M microscope (Zeiss) equipped with an AxioCam Mrm camera (Zeiss). Deconvolution of image stacks was undertaken using Axiovision release 4.7 or 4.8 software. Routine image manipulation was performed using FIJI and Adobe Photoshop.

### Solubility profile analysis

For solubility analysis of PfCoronin purified 3D7 *P. falciparum* merozoites were hypotonically lysed by re-suspending the merozoites in water supplemented with complete EDTA-free protease inhibitor cocktail (Roche). The samples were snap frozen in liquid N_2_ and incubated on ice for 10 min to thaw, releasing the cell contents. Water soluble and insoluble proteins were separated by ultracentrifugation at 1,00,000*g* for 30 min at 4°C (TLA100.2 rotor, Beckman Optima TL Ultracentrifuge, Beckman Coulter). Water insoluble fractions were further treated with Na_2_CO_3_ pH 11.5 for 1 h at 4°C. Carbonate soluble and insoluble fractions were isolated by ultracentrifugation as described. Samples were adjusted with 4 x reducing sample buffer (RSB) and subject to sodium dodecyl sulfate polyacrylamide gel electrophoresis (SDS PAGE) followed by Western blot analysis.

### Biosensor analysis

The amino analogue of PI(4,5)P2 (NH2-PI(4,5)P2 was synthesized with an ω-amino group on the sn-1 position of a saturated lipid side chain as described previously [45]. NH2-PI(4,5)P2 was then conjugated with Sulfo-NHS-biotin (Thermo Scientific) to enable immobilization onto the NeutrAvidin derivatized sensor surface according to a previously described protocol [46]. Experiments were performed using a Biacore 3000 biosensor (Biacore Life Sciences, GE Healthcare). Various concentrations of PfCor-N (2.6 μM, 1.3 μM, 650 nM, 325 nM, 162.5 nM and 81.2 nM) and PfADF1 (3.8 μM, 1.9 μM, 950 nM, 475 nM, 237.5 nM, and 188.8 nM) were injected over PI(4.5)P2, immobilized onto a CM5 sensor surface derivatized with NeutrAvidin using NHS/EDC chemistry (140RU immobilized) (Catimel 2013). A NeutrAvidin channel was used as the control. The reactivity of immobilized PI(4,5)P2 was assessed by injecting various concentrations of the GST-tagged Pleckstrin Homology domain of Phospholipase C, gamma 1 (GST-PLCδ-PH) (350, 175, 87.5, 43.8, 21.9 and 11 nM) [46].

Kinetic constants were derived from the resulting sensorgrams with BIAevaluation 4.1 software (Biacore Life Sciences, GE Healthcare) using Global analysis using a 1:1 Langmuir model that includes terms for mass transfer of analyte to the surface.

## Results

### Full length PfCoronin is unstable

**Figure 1:**
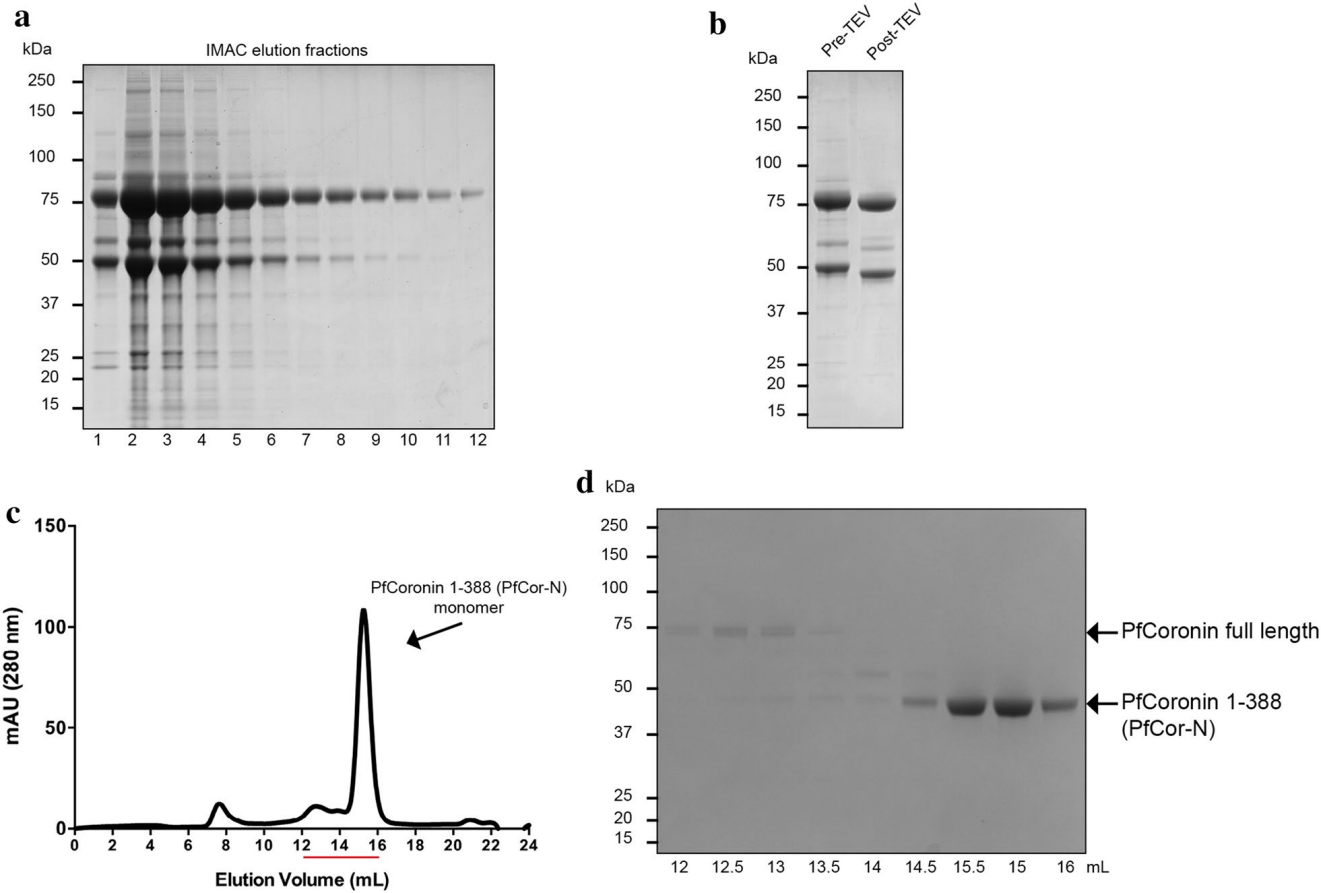
Purification of PfCoronin. **a** IMAC elution fractions 1–12. **b** PfCoronin pre- and post-removal of the N-terminal 6×His tag with TEV protease. **c** Size exclusion chromatography elution profile of PfCoronin. **d** Size exclusion chromatography elution fractions between 12 and 16 mL as indicated by the *red line* in (**c**) with PfCor-N eluting as monomer.

**Figure 2:**
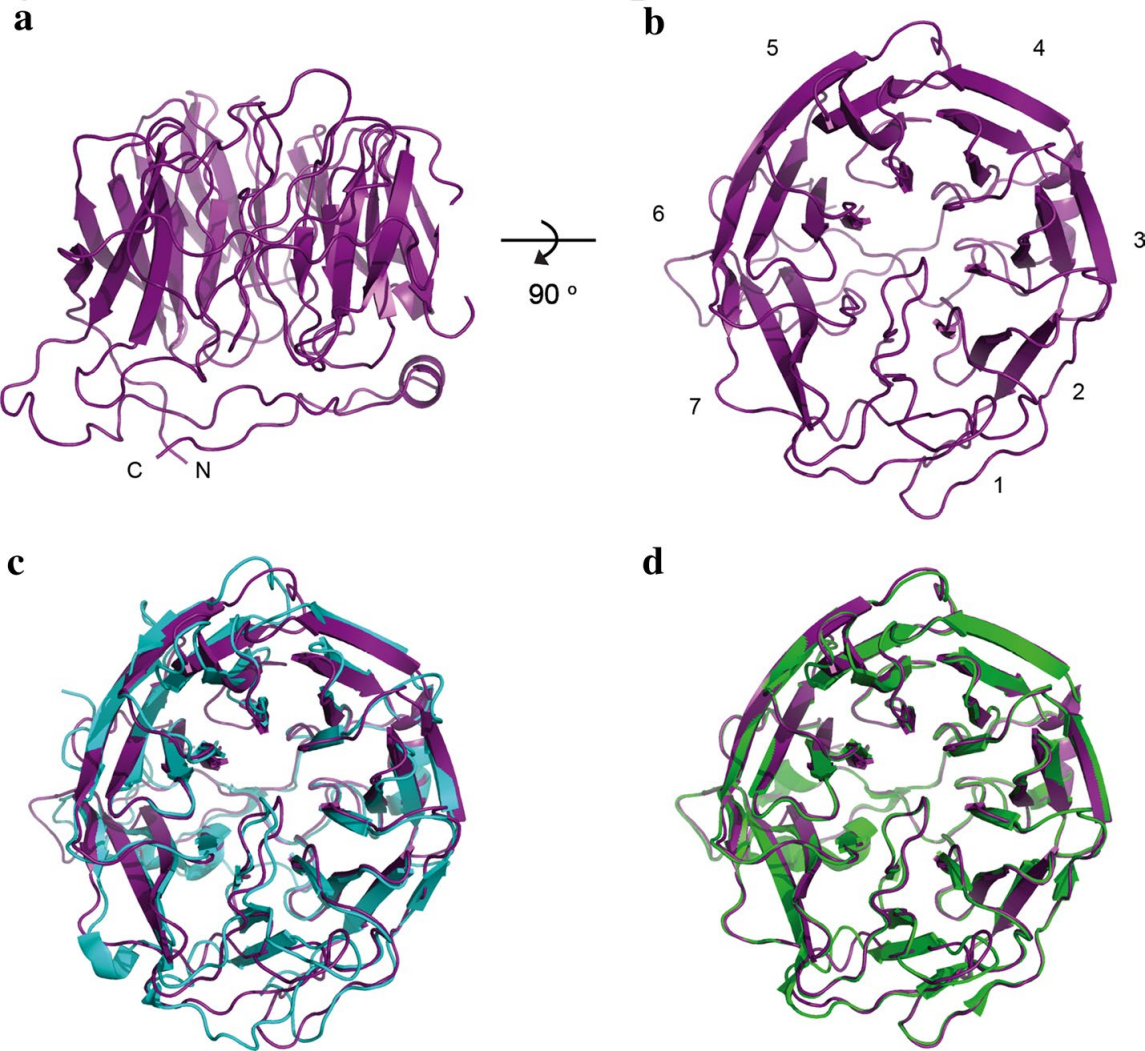
Homology model of PfCoronin. **a** *Side view* of homology model for PfCoronin generated using the I-TASSER server. **b** *Top view* of model. Potential blades within the propeller numbered 1–7 as per MmCoro1A and TgCoronin. **c** Alignment of PfCoronin homology model (*purple*) with MmCoro1A structure (2AQ5.pdb, [29]) (*blue*). **d** Alignment of PfCoronin homology model (*purple*) with TgCoronin structure (4OZU.pdb, [32]) (*green*).

To begin to explore the contribution of *P. falciparum* coronin (PfCoronin) to gliding motility, an N-terminally 6×His tagged PfCoronin was expressed and purified using a baculovirus expression system (Figure 1). Purification was performed according to an established method [29], consisting of immobilized metal affinity chromatography (IMAC), TEV cleavage to remove the His-tag and size exclusion chromatography. Throughout the purification process full-length PfCoronin (~69 kDa) rapidly degraded into a stable breakdown product of ~45 kDa (Figure 1). This was identified by mass spectrometry as the N-terminal portion of PfCoronin (amino acids 1–388) and agrees with the findings of others who observed a similar instability of purified murine coronin, which degraded from full length into a stable N-terminal breakdown product consisting of a seven bladed β-propeller domain [29]. Size exclusion chromatography demonstrated that PfCoronin (1–388), referred herein as PfCor-N, exists as a monomer in solution (Figure 1c). The degradation of predicted C terminal dimerization motifs precludes assessment of full length PfCoronin’s ability to form multimeric complexes. Structural modelling demonstrates a likely conservation of the N-terminal β-propeller structure with that in the murine and *T. gondii* coronin structures [29, 32], suggesting structural and functional conservation (Figure 2). Sequence comparison with the Salamun et al. structure revealed only minor divergences in the potential actin binding residues between Apicomplexan coronins and those in yeast Crn1 [32], hinting towards evolutionary conservation in the interaction between Apicomplexan coronins and their actins. Given the instability of the full length PfCoronin, all biochemical analyses were performed using the stable breakdown product, PfCor-N, containing the β-propeller domain, which contains the predicted conserved actin-binding regions.

### PfCor-N binds to F-actin

**Figure 3:**
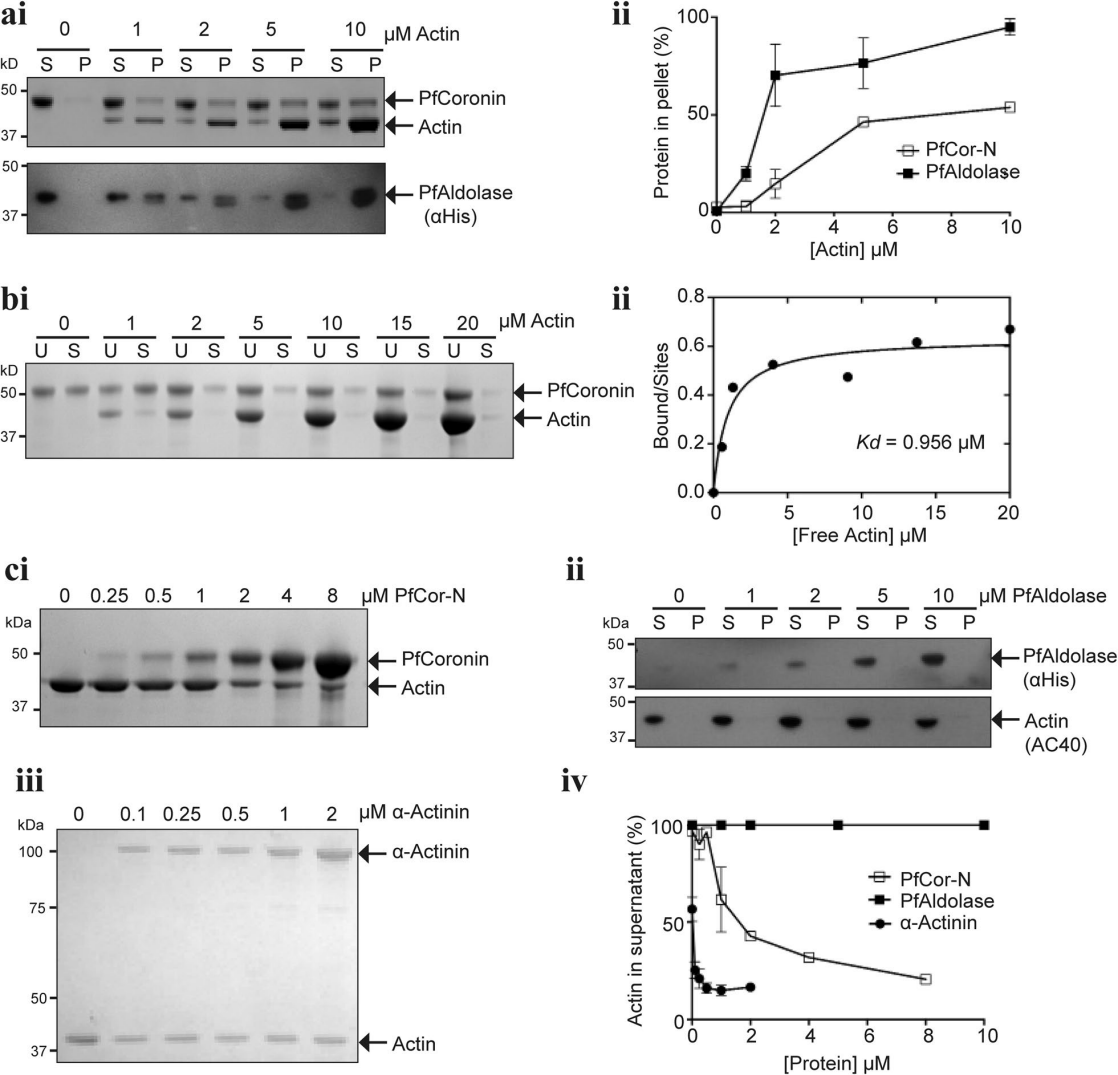
PfCor-N binds to and bundles F-actin. **ai** High speed sedimentation of PfCor-N (2 µM) or PfAldolase (2 µM) with 0–10 μM pre-assembled F-actin. Supernatant (S) and pellet (P) fractions shown for representative gel of PfCor-N (Coomassie stained) or PfAldolase (Western, probed with anti-His). **ii** Densitometry showing % protein co-pelleting with F-actin, values = mean ± SEM (n = 3). **bi** Supernatant depletion assay showing unspun (U) and spun (S) samples PfCor-N (2 μM) incubated with pre-assembled F-actin (0–20 μM) **ii** Densitometry of bound PfCor-N based on amount of free actin in solution post-centrifugation, providing a dissociation constant (*Kd*) = 0.956 μM. **c** Low-speed sedimentation assay of 2 uM F-actin assembled in presence of 0–10 μM PfCor-N (**i**), PfAldolase (**ii**) or α-Actinin with densitometry (**iv**) shown as % actin remaining in supernatant post-centrifugation, values = mean ± SEM (n = 3).

To investigate the interaction between PfCor-N and F-actin, sedimentation assays using purified rabbit skeletal muscle actin (RSMA) were preformed (Figure 3). PfAldolase was used as a positive control as a known F-actin binding protein [47]. The similarity in size between PfAldolase and actin required the use of western blots to differentiate specifically between these proteins (Figure 3ai). Both PfCor-N and PfAldolase co-pelleted with F-actin in a concentration dependant manner, indicating that each is able to efficiently bind F-actin (Figure 3a). To determine the affinity of the PfCor-N-F-actin interaction, an established supernatant depletion method was utilized [34]. PfCor-N binds F-actin with a *Kd* = 0.96 μM, a value between the range of *Kd*’s found among other type I coronins such as human variants Coronin 1A–1C (1A, 2.57 µM; 1B, 0.47 µM; and 1C 0.26 µM) [48] (Figure 3b). PfAldolase has been previously reported to bind F-actin with a *Kd* = 0.37 μM [47]. These results confirm PfCor-N as a verified F-actin binding protein.

### PfCor-N bundles F-actin into parallel bundles

Low speed sedimentation assays were used as a preliminary measure of F-actin bundling, where bundles being larger than individual actin filaments are able to sediment at lower speeds [49, 50]. Depletion of actin from the supernatant was, therefore, used as a measure of for bundling capability (Figure 3c). PfCor-N successfully depleted actin from the supernatant in a concentration dependant manner, indicating an ability to bundle F-actin (Figure 3ci). As positive control, α-actinin, a well-characterized F-actin crosslinking/bundling protein [51, 52] was also analysed, showing highly effective bundling at sub-micromolar concentrations (Figure 3ciii). PfAldolase, however, did not cause bundling even at the highest concentration tested (Figure 3cii), in contrast to reports of other species of aldolase bundling F-actin [8].

**Figure 4:**
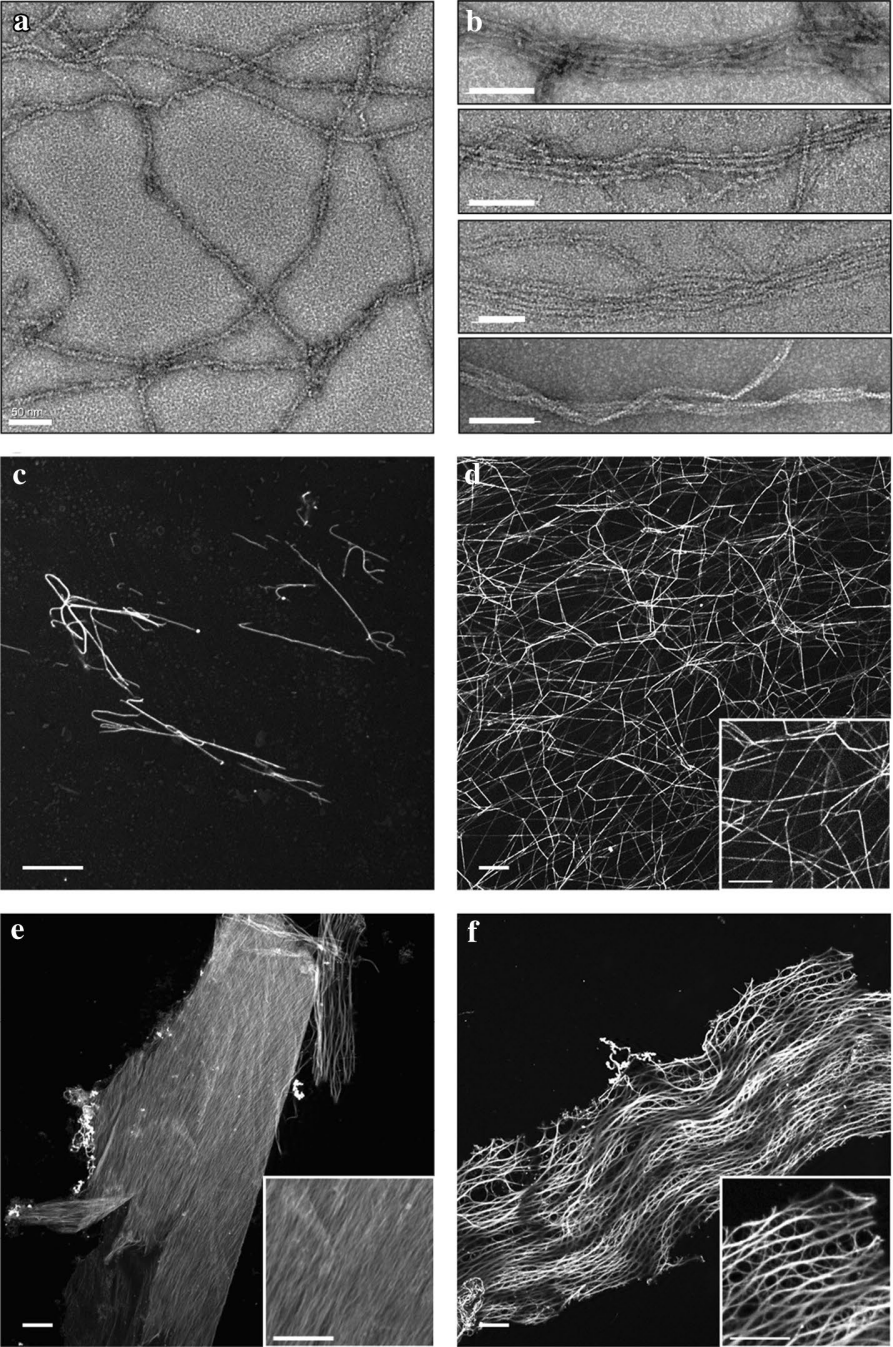
PfCor-N organizes F-actin into bundles and higher order sheets and networks. TEM of F-actin (2 μM) alone (**a**) or in presence of 0.2 μM PfCor-N (**b**, *top three panels*) and 1 μM PfCor-N (**b**, *lower panel*). *Scale bars* 50 nm (**a**), 100 nm (**b**). **c**–**f** Confocal micrographs of Phalloidin-488 labelled F-actin (1.5 μM) (**c**) alone, or in the presence of **d** 0.5 μM α-actinin, (**e**–**f**) 0.5 μM PfCor-N. *Scale bars* 10 μm.

To ensure that the results of the sedimentation analysis were the result of bundling and not protein aggregation samples were visualized by Transmission Electron Microscopy (TEM) to distinguish the formation of true filament bundles (Figure 4a, b). In the absence of PfCor-N, actin filaments were randomly settled across the field of view (Figure 4a). In contrast, filament bundles were observed in the presence of PfCor-N (Figure 4b). This is particularly noteworthy since, in other coronins, actin filament bundling has only been seen when the C-terminal oligomerization motifs are present [32, 53, 54].

Due to limitations of negative-stain TEM for imaging large, dense structures, a lower resolution confocal imaging approach using phalloidin-labelled F-actin was used to visualize higher order F-actin networks formed in the presence of the bundling/crosslinking proteins (Figure 4c–f). As expected, single actin filaments were visualized in the absence of bundling/crosslinking proteins (Figure 4c). In the presence of α-actinin, large networks of crosslinked F-actin appeared, similar in structure to previous reports (Figure 4d) [52, 55]. In the presence of PfCor-N however, two different structures emerged: extensive sheets of evenly distributed actin filaments and organized rows of cable-like structures (Figure 4e, f).

**Figure 5:**
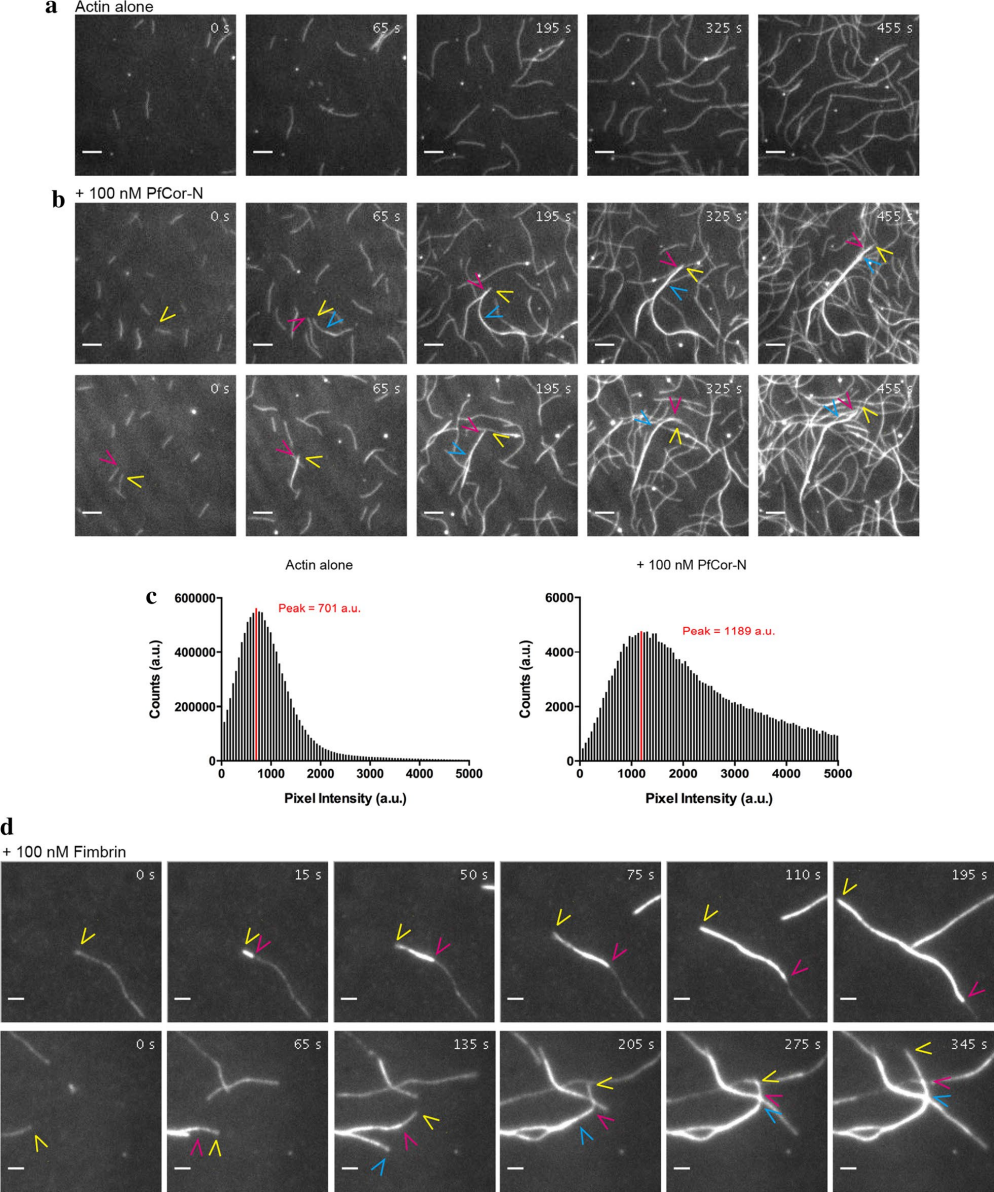
PfCor-N produces parallel actin bundles observed by TIRF microscopy. 2 μM Mg-ATP-actin with 1 μM Oregon Green Mg-ATP-actin (**a**) alone (**b**) + 0.1 μM PfCor-N. *Arrowheads* indicate growing ends of filaments. *Scale bars* 5 μm. **c** Pixel intensity quantification of **a** and **b** across 45 frames represented as measure of filament bundling. Actin alone pixel intensity mode = 701 arbitrary units (au) (*red line*). 100 nM PfCor-N pixel intensity mode = 1,189 au (*red line*). **d** Actin assembled in presence of 100 nM Fimbrin. *Top panel* antiparallel bundle, *bottom panel* parallel bundle. *Arrowheads* indicate growing ends of the filaments. *Scale bar* 15 μm.

Actin filaments are polarized, with elongation occurring predominantly at the barbed end of the filament. As such, bundles can either be parallel, where the barbed ends of the filaments are aligned and growing in the same direction, or antiparallel, where barbed ends are pointed away from each other and elongation occurs in both directions along the bundle. To distinguish parallel from antiparallel bundles the formation of actin filaments was observed in real time using total internal reflection fluorescence (TIRF) microscopy of Oregon Green labelled RSMA. In the absence of binding protein, actin filaments are seen to elongate randomly across the full field of view (Figure 5a; Additional file 1: Movie S1). In the presence of PfCor-N actin filaments can be seen being bundled together as they elongate (Figure 5b; Additional file 2: Movie S2). Bulk analysis of pixel intensity was used as a measure of filament bundling [50, 56, 57]. Actin alone showed a Gaussian distribution of pixel intensity, while in the presence of PfCor-N the pixel intensity is skewed to the right (Figure 5c). This shift is again indicative of filament bundling, wherein bundles have higher pixel intensities compared with single filaments [50].

Critically, tracking of the individual microfilaments elongating into a bundle demonstrated a clear directionality. In all cases, tracking the barbed end of each filament in the presence of PfCor-N showed evidence for exclusively parallel forming filament bundles (Figure 5b coloured arrows; Additional file 3: Movie S3 and Additional file 4: Movie S4). For comparison the bundling protein fimbrin was used, which is known to mediate both parallel and antiparallel bundles [50]. As expected, both types of bundles were visualized in the same experiment (Figure 5d; Additional file 5: Movie S5 and Additional file 6: Movie S6). Taken together, the biochemical and microscopy data suggest that PfCor-N is capable of both binding to F-actin and organizing filaments into parallel bundles, which can then organize into large, sheet and cable like structures.

### PfCoronin is expressed during schizogony and localizes to the periphery of merozoites before and during invasion

**Figure 6:**
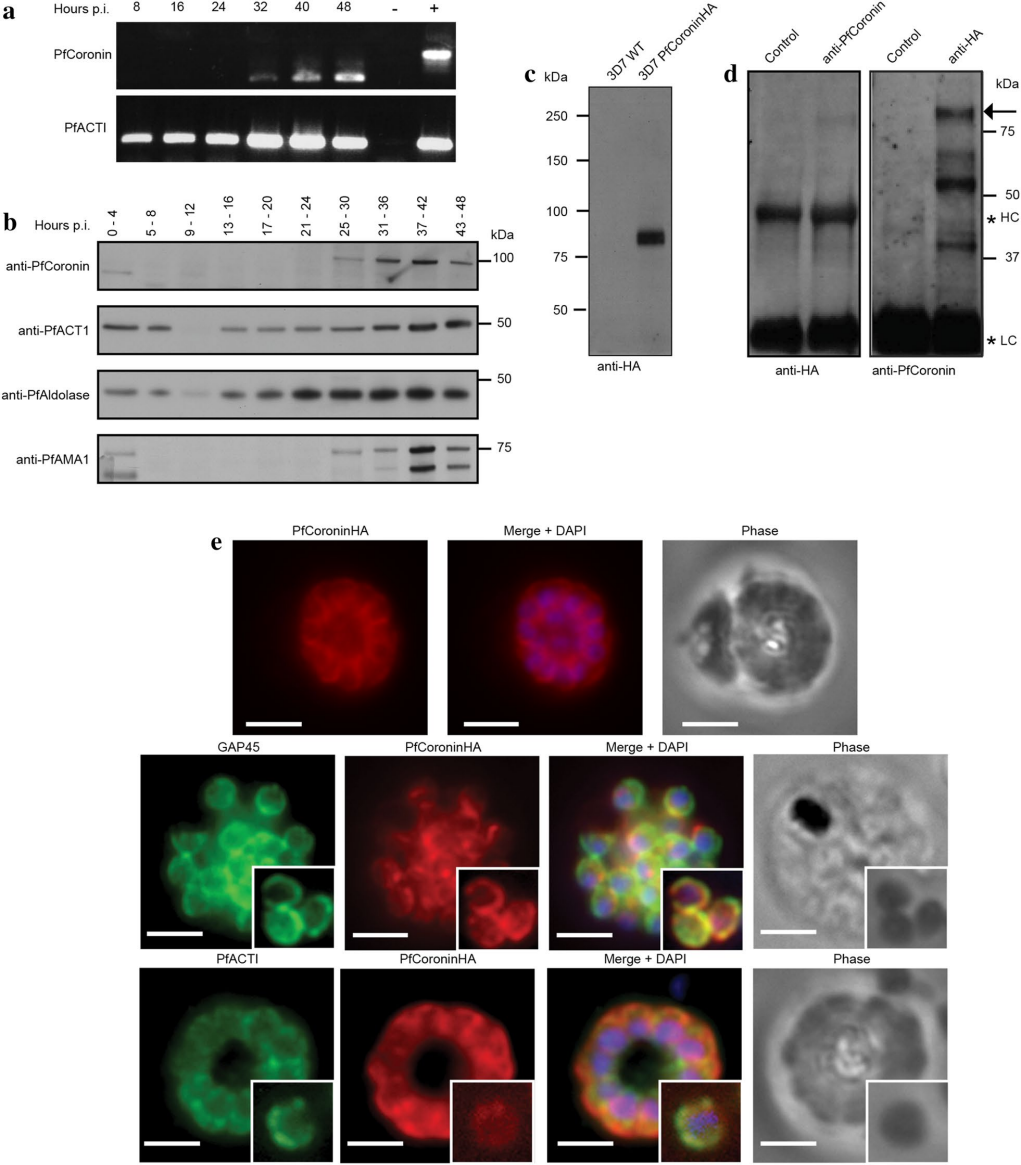
PfCoronin is expressed in schizonts/merozoites and is located at the periphery of mature merozoites. **a** RT-PCR of *P. falciparum* cDNA for *PfCoronin* and *PfACTI* genes across asexual life-cycle post invasion (pi). gDNA positive control (with introns). Negative control—no reverse transcriptase. **b** Western blots across asexual life-cycle post invasion (pi), probed with anti-PfCoronin, anti-PfACT1 (anti-Act239-253 (rabbit), [10]), anti-PfAldolase [40] and anti-PfAMA1 [60]. **c** Western blot WT 3D7 *P. falciparum* versus PfCoroninHA, probed with anti-HA. **d** Reciprocal western blots of immunoprecipitations from 3D7 PfCoroninHA schizont lysates with anti-PfCoronin bait, probed with anti-HA (*left panel*) or anti-HA bait, probed with anti-PfCoronin (*right panel*). Control lanes = WT 3D7 *P. falciparum*. *Arrow indicates* PfCoroninHA. *Asterisks* heavy and light chain cross-reactivity. **e** IFA of schizonts probed with anti-HA (Coronin, *red*), DAPI (nucleus, *blue*), anti-GAP45 (IMC, *green*) (*middle panel*) [40] or anti-PfACTI (IMC/cytosol *green*) (*bottom panel*). *Scale bar* 2 μm.

Given its potential to form filamentous macroscopic structures in vitro the spatial localization of native PfCoronin was explored in *P. falciparum* parasites to assess its potential role in the asexual life cycle. Evaluation of mRNA transcript levels by RT-PCR revealed that the gene for PfCoronin is transcribed late in intra-erythrocytic development, with a peak around 40–48 h, corresponding to late schizonts/maturing merozoites (Figure 6a), consistent with predicted levels [58]. Of note, the size differential between genomic and mRNA PCR products conforms to the predicted presence of two introns in the native gene [59], totalling 336 base pairs, which causes a visible size shift of the band compared to a gDNA control (Figure 6a). In parallel, analysis of the PfACTI gene showed transcription levels across the asexual lifecycle.

To confirm this expression profile for the transcribed protein, a polyclonal rabbit antibody was generated against the N-terminal portion of PfCoronin (residues 1–388, anti-PfCoronin) and validated for use in immunoblot analysis (see below). Analysis of parasite material by immunoblot probed with anti-PfCoronin confirmed a peak of protein expression late in the asexual lifecycle, in accordance with the level of the gene transcript (Figure 6b). The same samples were also probed with anti-PfACTI (anti-Act239-253 (rabbit), [10]), anti-PfAldolase [40] and anti-PfAMA1 [60]. PfACTI was detected across the time-course with a broad peak over the later stages of the lifecycle. PfAldolase levels were consistent with those of actin. PfAMA1, in its processed and unprocessed forms (80 kDa/62 kDa respectively, [61]), showed a distinct peak in late schizonts and early in invasion, consistent with its known role as an invasion ligand [62] (Figure 6b). The similarity between the expression profiles of PfCoronin and PfAMA1 is consistent with PfCoronin playing a role in blood stage merozoite invasion of the erythrocyte, a process dependent on active actomyosin motility [13].

Given a variable background in immunofluorescence microscopy assays (IFA) with anti-PfCoronin due to background non-specific labelling, endogenous tagging of the native *coronin* gene (PF3D7_1251200) with a C-terminal 3× haemagglutinin (HA) tag was attempted. Expression of PfCoroninHA was confirmed by Western blot of schizont lysate probed with anti-HA (Figure 6c), revealing a dominant band running above the 75 kDa marker absent in WT controls. This is moderately higher than the predicted molecular weight of PfCoroninHA (~72 kDa), but could be indicative of membrane interactions or net negative charge that may effect protein migration through SDS. As validation, immunoprecipitation of tagged PfCoronin from late-stage schizont was performed using both anti-PfCoronin and anti-HA, with probing using the reciprocal antibody (Figure 6d). In both combinations the same band above 75 kDa was found, which was absent in pre-immune serum control. Taken together, this data validated the antibody and confirmed incorporation of the C-terminal HA-tag on PfCoronin. Visualization of PfCoroninHA in parasites in late stage schizonts revealed a distribution predominantly at the periphery of the merozoites within the schizont (Figure 6e). Co-labelling of schizonts with antibodies against PfGAP45, a myosin motor accessory proteins that is bound to the outer face of the IMC and plasma membrane [5], and PfACTI, which is known to concentrate to the parasite periphery [10], demonstrated a broadly consistent spatial localization of both proteins within the pellicular space (Figure 6e).

**Figure 7:**
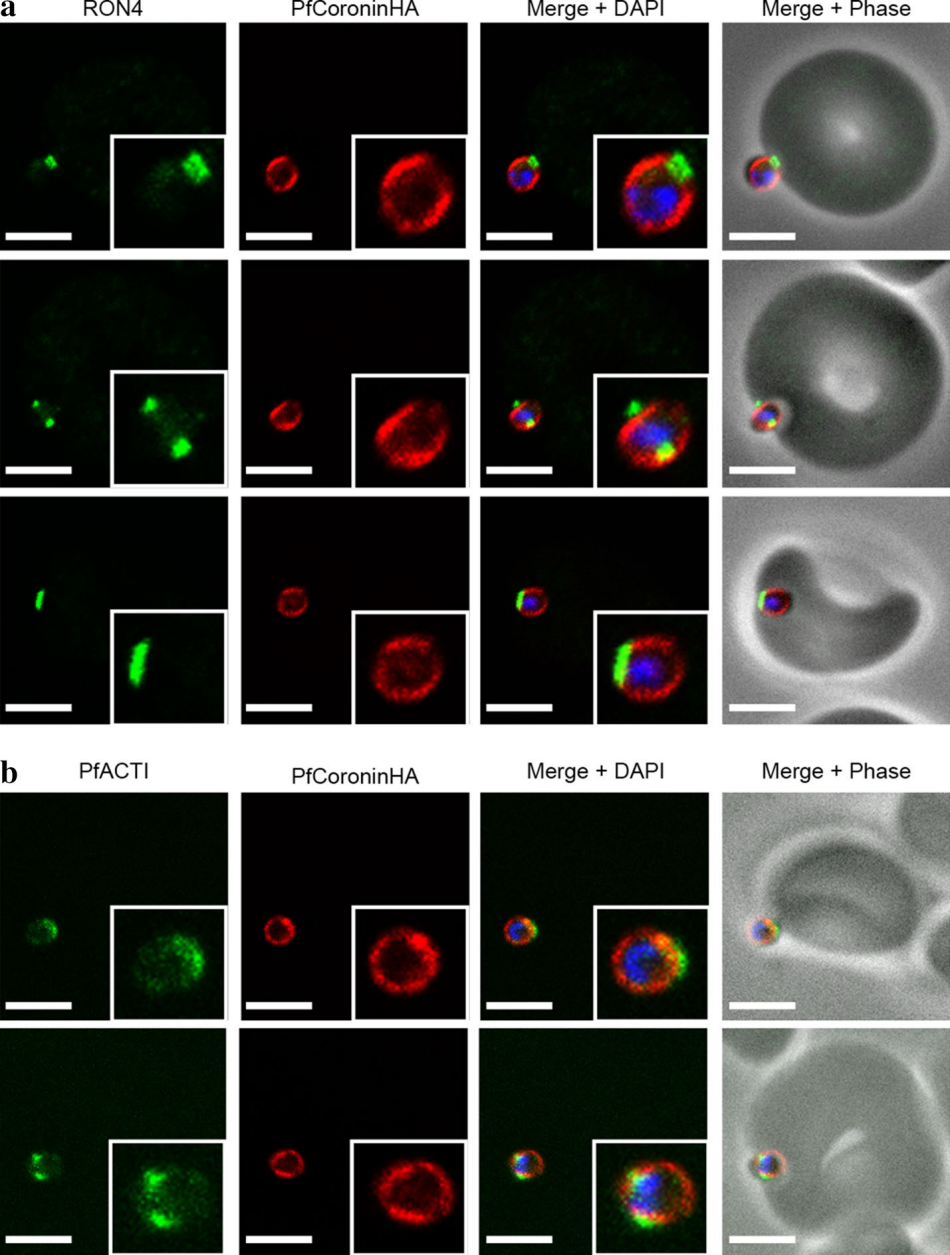
PfCoroninHA remains peripherally located in merozoites throughout erythrocyte invasion. **a** Invading merozoite IFA early (*top panel*), mid (*middle panel*) and late (*bottom panel*) in invasion. Labeling is anti-RON4 (tight junction, *green*), anti-HA (Coronin, *red*) and DAPI (nucleus, *blue*). *Scale bar* 2 μm. **b** Invading merozoite IFA early (*top panel*) and midway (*bottom panel*) through invasion. Labeling is anti-PfActin (IMC/cytosol, green), anti-HA (Coronin, *red*) and DAPI (nucleus, *blue*). *Scale bar* 2 μm.

Many proteins that show a pellicular localization in schizonts will re-distribute during merozoite invasion, indicating their involvement in the tight junction or the acto-myosin motor, such as PfAMA1 and PfACTI [10, 41]. To determine if PfCoroninHA redistributed during invasion, invading merozoites captured early, mid and late in the invasion process were co-labelled with RON4, a marker of the tight junction ring (Figure 7a) [41]. The localization of PfCoroninHA, in contrast to expectations, was stable throughout the invasion process with no major redistribution occurring. Co-localization of PfCoroninHA with PfACT1 during invasion did not reveal any striking correlation with that of F-actin (the major epitope of the Act239-253 antibody) [10] (Figure 7b). Thus PfCoronin appears to occupy a fixed localization at the pellicle of invasive merozoites during their entry into the erythrocyte.

### Membrane association of PfCoronin is likely via PI(4,5)P_2_ binding

**Figure 8:**
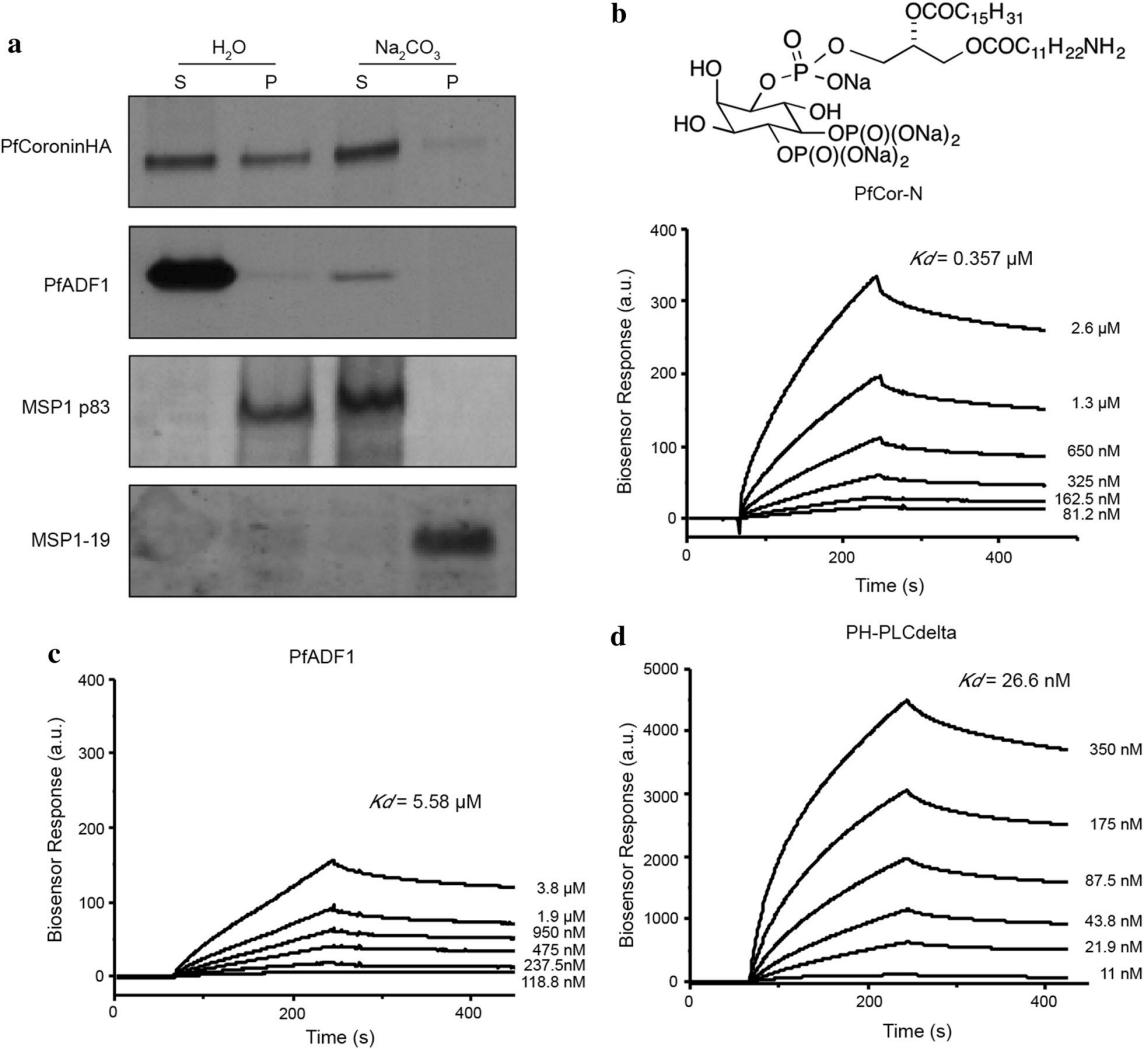
Membrane association of PfCoroninHA is mediated by PI(4,5)P_2_. **a** Western blot of PfCoroninHA. *Lanes 1* and *2* supernatant (S) and pellet (P) post hypotonic lysis. *Lanes 3* and *4* S and P post Na_2_CO_3_ treatment. *Top panel* probed with anti-HA (Coronin). *Second panel* probed with anti-PfADF1 [63]. *Third panel* probed with anti-MSP1p83 (non-membrane associated protein). *Fourth panel* probed with anti-MSP1-19 (membrane associated GPI anchor). **b**–**d** SPR sensograms of (**b**) PfCor-N (**c**) PfADF1 and (**d**) PH-PLCdelta binding to immobilized PI(4,5)P_2_, shown above panel **b**. Concentrations of each analyte displayed by curves. Determined *Kd*s = inset in graph.

Since PfCoronin lacks any obvious post-translational lipidation motifs or a transmembrane domain, the nature of the peripheral localization of PfCoroninHA in merozoites was explored. Towards this, purified merozoites were subjected to hypotonic lysis followed by centrifugation to separate cytoplasmic proteins from those that are membrane associated. The membrane-associated fraction was then treated with Na_2_CO_3_ to allow discrimination between integral membrane proteins and those that are associated with membranes. Western blot analysis demonstrated that approximately 40% of PfCoroninHA is membrane-associated (Figure 8a). As controls, the samples were also probed with antibodies against PfADF1, a soluble cytoplasmic protein [63], GPI-anchored MSP1-19 which remains in the pellet [64], and MSP1p83, a cleaved membrane associated product which is lost to the supernatant post carbonate treatment (Figure 8a) [64]. PfCoroninHA was found in the carbonate-dependent supernatant confirming that whilst not directly integrated into the membrane, it is bound via alternate means.

Coronin proteins from other eukaryotes have been shown to associate with cellular membranes via interaction with phosphatidylinositol 4,5-bisphosphate (PI(4,5)P_2_) [65]. This was tested using recombinant PfCor-N, measuring its affinity for PI(4,5)P_2_ via Surface Plasmon Resonance (SPR) with chip-bound amino-terminal PI(4,5)P_2_. As controls, PfADF1, a *Plasmodium* actin binding protein that does not have strong affinity for phosphatidylinositol derivatives [63], and the PH domain of Phospholipase C delta (PH-PLCdelta), a well defined PI(4,5)P_2_ binding protein [66] were also tested. Calculation of the *Kd* for each protein revealed values of 0.36 μM for PfCor-N, 5.5 μM for PfADF1 and 27 nM for PH-PLCdelta (Figure 8b–d). This establishes that PfCor-N binds to PI(4,5)P_2_ with sub-micromolar affinity. Combined with IFA determined localization of PfCoroninHA, this data supports the notion that PfCoronin associates with the parasite plasma membrane likely via interaction with resident phospholipids. Placement in this compartment with other components of the motor complex combined with biochemical data is consistent with PfCoronin playing an organizing role for actin during *Plasmodium* blood stage cell motility.

## Discussion

Apicomplexan gliding and host cell invasion are known to be reliant on a conserved actomyosin motor that generates the force necessary for directional motility. This motor requires dynamic actin filaments, anchored in the parasite pellicular space, to provide a track along which myosin can engage and drive forward cell motion. The actin filaments at the core of the motor are short, unstable and highly dynamic with unusual kinetics [67–73]. Furthermore, they show no evidence of forming any ordered actin structure within the parasite cell [9, 10]. Indeed, current evidence suggests that the majority of actin in cells is monomeric, with only ~2% predicted to be incorporated into filaments [74]. Indeed, their highly dynamic nature is essential for functional motility, as treatment of parasites with actin inhibitors impedes host cell invasion and gliding motility [13, 14, 75, 76]. Given such dynamics there is still a major gap in current understanding as to how directional motility, specifically the provision of oriented actin microfilament tracks for myosin, is achieved.

Until recently, it was believed that part of the process of motor engagement, and potentially a major organizing component, came from anchoring of actin filaments to secreted adhesins via the tetrameric enzyme fructose 1,6 bisphosphate aldolase [7]. Indeed several adhesins from the thrombospondin related anonymous protein (TRAP) family [7, 40, 77] and other unrelated proteins [47, 78] have been independently linked to aldolase. However, recent evidence suggests that whilst the binding may occur readily in vitro (via pull downs) or in the native cell, the interaction in vivo is not a functional requirement for normal motility. Rather it is primarily involved in energy metabolism in the parasite cell [11]. Thus whilst its role in recruiting energy sources to regions of motor activity may still be critical to motility, it may not play any organizing role in the motor. These observations highlight the clear lack in understanding about the entire organization of the motor complex and how it leads to directional force and movement. Evidence presented here suggests coronin as a first organizing factor that links F-actin with the parasite plasma membrane, arranging these into parallel bundles and as such contributing directly to directed gliding motility.

Here, evidence clearly describes the ability of the β-propeller domain of PfCoronin to bundle actin filaments together using bulk biochemical assays and multiple microscopic techniques. The filament-bundling capacity is somewhat surprising, as previous reports of bundling by other coronins required homo-oligomerization mediated by the C-terminal CC domain to bring multiple actin filaments together into a bundle [30, 32, 53, 79]. However, recent mutational studies have identified multiple binding sites for F-actin on coronin [48, 49], which form a ridge that spans the length of the β-propeller domain [49]. It has been postulated that these multiple binding sites could be interacting with two or more actin monomers within the filament [49, 80], or perhaps, given the results of this study, with two or more monomers from different filaments. Further mutational analysis of the actin binding sites in PfCoronin will be essential to address this phenomenon of F-actin bundling by monomeric coronin.

In vivo, PfCoronin was shown to display a peripheral localization, consistent with the pellicle space (Figure 6e). This data, in combination with a peak of protein expression in maturing and invading merozoites (Figure 6a, b), alludes to a role for PfCoronin in the invasion process. However, the consistent spread of PfCoronin at the parasite periphery during invasion suggests that this role is not limited to linking actin filaments to the plasma membrane at regions of known motor engagement (Figure 7). Indeed, the interaction of PfCor-N with PI(4,5)P_2_ in vitro suggests that the linkage between actin and the membrane may be more direct than previously envisaged. Rather than actin linking via exclusive interactions with tetrameric aldolase to the tails of secreted surface adhesins [7] the entire track for myosin force generation may be bound to the plasma membrane or sub-domains within it. Whilst the caveats of PfCor-N interactions with vertebrate actin need to be verified with a reliable source of correctly folded *Plasmodium* actin, if validated, the bundling ability of the protein combined with its in vivo distribution would suggest that native PfCoronin may be constantly organizing actin into ordered arrays underlying the plasma membrane, which are temporarily stabilized during motor engagement, permitting any associated adhesin to facilitate the transmission of motor force. Such a scenario would make the apicomplexan actomyosin motor look more muscle-like with an organized face at the IMC side of the pellicular space dedicated to myosin motor organization, and at the plasma membrane arrayed patches of parallel-bundled actin filaments ready for myosin engagement. Further mutational, optical and detergent extraction approaches may provide insights into this organization in support of such a model.

Although PfCoronin may be providing an organizing template for motility, there is still a need to explain how myosin motor force is directed and how actin filament polarity is determined within the context of the pellicular space. A portion of directionality determination may fall to the other actin regulators, such as the formins [18]. Further work in this area is clearly needed. In addition, the exact contribution of PfCoronin to parasite motility, the effect PfCoronin binding to actin (and importantly native actin) has on the myosin motor and further comprehensive genetic dissection in *P. falciparum*, via knockout, conditional knockdown or expression of domain deletions, will be important for understanding the overall regulation of the spatial organization of actin in the parasite pellicle, and consequently the mechanics of host-cell invasion and directional gliding motility.

## Conclusion

In summary, evidence is presented demonstrating actin filament bundling by PfCoronin in vitro that, combined with in vivo imaging data and phospholipid binding, supports a role for PfCoronin being an important effector for organizing actin filaments in the invasive malaria parasite. If validated this would open up the possibility that Coronin across apicomplexan parasites, and across *Plasmodium* life cycle stages, may be a key organizing force for directional actin filaments and by extension gliding motility in these key human pathogens.

## Additional files

Additional file 1:
**Movie S1.** TIRF OG-Actin alone. The movie demonstrates TIRF microscopy of actin filaments polymerising alone. Additional file 2:
**Movie S2.** TIRF OG-Actin plus PfCoronin, full view. The movie demonstrates TIRF microscopy of actin filaments polymerising in the presence of PfCoronin-N terminal protein. Additional file 3:
**Movie S3.** TIRF OG-Actin plus PfCoronin, zoom view I. The movie demonstrates TIRF microscopy of actin filaments polymerising in the presence of PfCoronin-N terminal protein zoomed into demonstrate a first example of parallel filament-bundling. Additional file 4:
**Movie S4.** TIRF OG-Actin plus PfCoronin, zoom view II. The movie demonstrates TIRF microscopy of actin filaments polymerising in the presence of PfCoronin-N terminal protein zoomed into demonstrate a second example of parallel filament-bundling. Additional file 5:
**Movie S5.** TIRF OG-Actin plus Fimbrin, parallel filaments. The movie demonstrates TIRF microscopy of actin filaments polymerising in the presence of purified Fimbrin, demonstrating parallel bundling of filaments. Additional file 6:
**Movie S6.** TIRF OG-Actin plus Fimbrin, anti-parallel filaments. The movie demonstrates TIRF microscopy of actin filaments polymerising in the presence of purified Fimbrin, demonstrating anti-parallel bundling of filaments.

## Abbreviations

PfCor-N: PfCoronin residues 1-388
ADF: actin depolymerizing factor
RSMA: rabbit skeletal muscle actin
TIRF microscopy: total internal reflection fluorescence
IMC: inner membrane complex
